# Epigenetic and Neurological Impairments Associated with Early Life Exposure to Persistent Organic Pollutants

**DOI:** 10.1155/2019/2085496

**Published:** 2019-01-14

**Authors:** Nathalie Grova, Henri Schroeder, Jean-Luc Olivier, Jonathan D. Turner

**Affiliations:** ^1^Immune Endocrine Epigenetics Research Group, Department of Infection and Immunity, Luxembourg Institute of Health, 29 rue Henri Koch, L-4354 Esch-sur-Alzette, Luxembourg; ^2^Calbinotox, Faculty of Science and Technology, Lorraine University, Campus Aiguillettes, B.P. 70239, 54506 Vandoeuvre-lès-Nancy, France

## Abstract

The incidence of neurodevelopmental and neurodegenerative diseases worldwide has dramatically increased over the last decades. Although the aetiology remains uncertain, evidence is now growing that exposure to persistent organic pollutants during sensitive neurodevelopmental periods such as early life may be a strong risk factor, predisposing the individual to disease development later in life. Epidemiological studies have associated environmentally persistent organic pollutant exposure to brain disorders including neuropathies, cognitive, motor, and sensory impairments; neurodevelopmental disorders such as autism spectrum disorder (ASD) and attention-deficit hyperactivity disorder (ADHD); and neurodegenerative diseases including Alzheimer's disease, Parkinson's disease, and amyotrophic lateral sclerosis (ALS). In many ways, this expands the classical “Developmental Origins of Health and Disease” paradigm to include exposure to pollutants. This model has been refined over the years to give the current “three-hit” model that considers the individual's genetic factors as a first “hit.” It has an immediate interaction with the early-life exposome (including persistent organic pollutants) that can be considered to be a second “hit.” Together, these first two “hits” produce a quiescent or latent phenotype, most probably encoded in the epigenome, which has become susceptible to a third environmental “hit” in later life. It is only after the third “hit” that the increased risk of disease symptoms is crystallised. However, if the individual is exposed to a different environment in later life, they would be expected to remain healthy. In this review, we examine the effect of exposure to persistent organic pollutants and particulate matters in early life and the relationship to subsequent neurodevelopmental and neurodegenerative disorders. The roles of those environmental factors which may affect epigenetic DNA methylation and therefore influence normal neurodevelopment are then evaluated.

## 1. Introduction

Early life is a critical period for human development, determining lifelong patterns of health and disease. The work of David Barker in the 1980s and 90s clearly identified the period from conception to age 2 y (the first 1000 days) as one of the key determinants of an individual's lifelong health trajectory [1]. So far, there has been considerable success in documenting these health inequalities. Indeed, strong epidemiological links have been established between measurements of suboptimal early-life conditions and a number of disease phenotypes. Most convincingly, poor early-life conditions have been epidemiologically associated with a series of lifelong, proinflammatory phenotypes, an increased risk for autoimmune and allergic disorders, and both physical and mental disorders. There have now been numerous cohort studies that have observed associations between the early-life environment and an increased risk of cardiovascular diseases, type 2 diabetes, allergies and asthma, autoimmune diseases such as multiple sclerosis and rheumatoid arthritis, migraine, obesity, and psychiatric disorders [2–6]. The underlying molecular mechanisms are starting to be addressed, and it is now evident that psychosocial exposures induce lifelong changes in fundamental identity of immune cells [7, 8].

Although this prior work has somewhat focussed on diseases with a large inflammatory basis, this time period may actually be as, if not more, important in many neurological diseases. As early life is of critical importance for brain maturation [9], numerous environmental factors occurring during this time can significantly affect its long-term functionality with potent lifelong health consequences. It is now clearly established that both the density/connectivity of neurons and the ability to use alternative networks, i.e., anatomical and functional organisation, respectively, are susceptible to changes in the early-life environment, leading to potential neurological disorders [10]. Indeed, there is clear evidence that the early-life environment is primordial in determining an individual's susceptibility to Alzheimer disease (AD) [11]. There is also evidence, although less well developed, for a similar link to Parkinson's disease (PD) [12]. However, correctly ascribing eventual brain disorders in adulthood to events occurring during early life remains somewhat challenging due to the very long latency. A developmental origin for neurological disorders such as AD and PD has been suggested for many years [11–16]. As our understanding of the “developmental origins of health and disease” has increased, it has become possible to track individual neurodevelopmental “trajectories” [17, 18]. However, what is still unknown is the aetiological agent, or agents, acting during early life.

**Figure 1:**
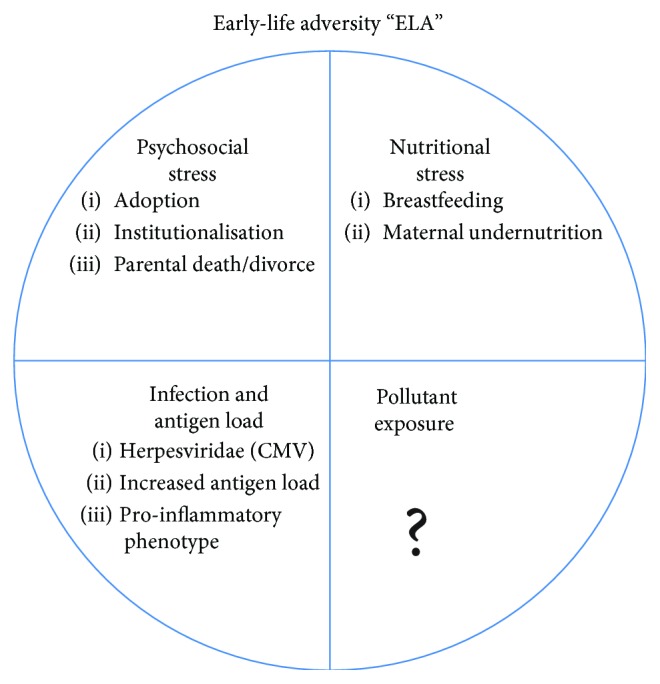
Epigenetically acting aspects of the early-life exposome. The early-life exposome has been divided into four key elements: psychosocial stress, nutritional stress, immunological stress, and exposure to pollutants.

Early-life adversity (ELA) is an umbrella that covers a mix of different, but concurrent types of exposure. ELA consists of four principal types of exposure: nutrition, psychosocial elements, infectious agents, and environmental pollutants (Figure 1). The difficulty lies in determining exact roles of each of the four exposure types as they are clearly intertwined. Indeed, commonly used measures of early-life adversity cover a wide spectrum of factors from well-defined exposure conditions (particles, pollutants, substances of abuse, etc.) to the more insidious, less uncertain, low socioeconomic status (SES). For instance, low SES includes the exposure to a persistent background of (i) increased environmental pollutants/irritants; (ii) financial and psychosocial stressors, with associated lifestyle factors such as smoking, alcohol consumption, and BMI, and (iii) an increased immunological burden with higher exposure to pathogens, antigens, and allergens. In addition, exposure is predominantly the result of a complex set of events and molecules, and not a single one. This has resulted in the “triple jeopardy hypothesis” where individuals that are raised under low SES conditions not only face an increased exposure to environmental hazards such as air pollution but also have increased psychosocial stress such as discrimination, chronic stress leading to poorer health, and health disparities that are driven by many other environmental factors [19].

There is now a small, but growing, literature exploring the influence of the early-life environment, associating exposure with neurological and neurodevelopmental conditions including AD, PD, autism spectrum disorders (ASD), and attention-deficient hyperactive disorder (ADHD). Although the aetiology of these diseases is still uncertain, the role of the early-life environment is slowly starting to be understood and accepted as a genuine risk factor [20].

Traditionally, neurotoxicology has concentrated on the effects of exposure on the fully developed, mature, and adult, although this is starting to change. Proven and potential neurotoxic substances include heavy metals, organic solvents, persistent organic pollutants (polychlorobiphenyls (PCBs), polychlorodibenzo-para-dioxines (PCDD), and polychlorodibenzofuranes (PCDF)), plastic exudates (bisphenol A (BPA) and phthalates), pesticides, brominated flame retardants (BFRs), and polycyclic aromatic hydrocarbons (PAHs) [21, 22]. Some are currently classified among 183 substances of very high concern (SVHC) in Annex XIV of the REACH European regulation defined by the European Chemical Agency (ECHA) [23]. BPA was recently added to the list of SVHC due to its endocrine-disrupting properties. However, our growing awareness of the importance of exposure during sensitive life-periods is reflected in the recent categorisation of hexabromocyclododecane (HBCDD, a BFR) and all its major diastereoisomers as an SVHC [23], as it was shown to be especially toxic to pregnant women, foetuses, and newborn babies. Nevertheless, due to a large number and variety of persistent chemicals in the environment, the knowledge of their respective neurotoxic potential unfortunately remains very limited to date.

There have been no concerted efforts to determine the individual effects of these four key early-life components so far; however, an increased number of epidemiological studies have associated exposure to persistent organic pollutants (POPs) with subsequent neurological impairment. In this review, we focus on the “empty” segment of the early-life adversity circle. We examine the effect of exposure to POPs and particulate matters in early life and the relationship to subsequent neurodevelopmental and neurodegenerative disorders. The role of those environmental factors which may affect epigenetic DNA methylation and therefore influence normal neurodevelopment is then evaluated.

## 2. Foetuses, Newborn Babies, and Toddlers: Particularly Vulnerable Populations

### 2.1. Vulnerability of Children to Chemical Exposure

Children are vulnerable to direct exposure caused by industrial use of chemical pollutants (e.g., pesticides used for crop treatment) and indirect exposure to these agents coming from polluted air or contaminated water and food, which is also prevalent. Hand-to-mouth contact, involving toddlers and infants, can also be assimilated to an additional route of exposure since it involves both ingestion and dermal absorption. Indeed, ingestion of dust was found to be one of the major contamination routes in toddlers (75%), who spend more time playing on the floor and putting their hands into their mouth. As a consequence, the daily intake of organic pollutants such as HBCDD was estimated to be higher in toddlers (400 to 1500 ng/day) than in adults (130 to 330 ng/day) [24, 25]. Similarly, daily intake by breast-fed infants of 2,3,4,7,8-PeCDD and PCB-126 was estimated in Korean mother-child diads at 85 TEQ pg/kg/day. This concentration was 20 times higher than that defined by the World Health Organization for adults [26]. There are several explanations for this increased exposure of infants. Firstly, due to the fact that children consume more food and more water per unit of body weight and present a higher ratio of body surface area to volume, they are exposed to higher concentration levels of POPs than individuals at adulthood [27]. Secondly, foetuses and newborn babies rank at the highest level of the food chain as they obtain nutrients required for their development through the mother's body, whether during pregnancy or breastfeeding [27]. Finally, it is now accepted that the physiology and metabolism of a child differ from adults and are somewhat immature. Consequently, pollutants are less detoxified and more highly concentrated in the body [28]. From conception until the end of puberty, physical growth and functional maturation of the body happen at a differentiated but constant rhythm [29]. POP exposure during these vulnerable windows of the child's life could adversely impact these dynamic processes and provoke irreversible damage which may become evident rapidly or much later in life [29]. Therefore, physiological and behavioural differences observed between children/infants and adults may account for the differential pattern of exposure and the subsequent physiological response.

### 2.2. Vulnerability of the Developing Brain

Although the common environmental pollutants differ wildly in their chemical properties, one common aspect is their lipophilicity and their strong affinity for lipid-rich tissues [30]. Since the brain is the principal lipid-rich tissue and one of the main fat depositories in the developing foetus, most of these pollutants are channelled there in disproportionate quantities, once they have crossed the placenta. Brain development is an extraordinarily complex phenomenon which is initiated in early gestational stages and continues for several years postnatally [31]. Within this period, cellular processes such as neurogenesis, migration, neuronal differentiation, synaptogenesis, myelination, apoptosis, and synaptic plasticity must take place within a rigorously controlled time frame in which each neurodevelopmental stage must start on schedule and follow a well-determined sequence [31]. Within this complex schedule, there are therefore windows of susceptibility to environmental insults which have no counterpart in the mature nervous system or in any other organs. If a developmental process in the brain is halted or inhibited, there are few possibilities for potential repair later on, and the consequences may be permanent [27, 31–33]. Unfortunately, the developing brain is poorly protected from chemical exposure during foetal and early postnatal life despite the presence of two important biological barriers: the placenta and the blood-brain barrier (BBB). It would appear that the placental barrier is somewhat porous and a significant number of POPs cross the placental barrier and enter the foetal blood stream. Levels of fifteen PAHs measured in ninety-five mother-child diads were significantly higher in cord blood sera than in maternal sera [34]; similarly, metabolite levels were higher in cord blood suggesting active foetal PAH metabolism and slow metabolite clearance from the foetal-placental compartment [35]. Although the BBB provides an additional critical layer of protection against many chemicals, its immaturity throughout early life leads to increased vulnerability of the brain to detrimental effects of environmental chemicals during key stages of its development [36].

These physiological differences suggest that the developing brain has a much greater chance of exposure to environmental pollutants, including chemicals that may be excluded by the mature adult BBB [37]. The concurrent porosity of these two barriers in early life has been demonstrated for a series of PAHs and their principal metabolites. After exposure of pregnant dams, both PAHs and their metabolites were observed in brains of the dams [38] and the F1 generation at PND0 [39] confirming that they pass the placenta and foetal BBB. These results are in line with previous work performed on rats which suggested storage of benzo[a]pyrene and its respective metabolites in cerebral tissues of pups exposed during gestation [40–43]. This in utero exposure is further compounded by the presence of PAH in breast milk and its subsequent transfer to pups [39]. Taken together, these data highlight that PAHs and/or their metabolites can be transferred to the brain throughout this particularly sensitive neurodevelopmental window. Furthermore, because of the developmental plasticity of neuronal networks, the brain remains susceptible to such environmental variations for several years after birth [27, 44]. This period of heightened vulnerability for the developing brain extends from the first days of conception to early childhood.

## 3. Early-Life Environmental Exposure and Epigenetic and Neurological Impairments in Children

### 3.1. ADHD

Attention-deficit/hyperactivity disorder (ADHD) is “a common, long-lasting and treatable childhood psychiatric disorder, characterised by a pattern of developmentally inappropriate inattention, motor restlessness, and impulsivity that affects approximately 3-7% of school-aged children” [45, 46]. Meta-analysis of 175 studies estimated worldwide prevalence of ADHD in children below age 18 to be around 7.2% [47]. Although the heritability of ADHD was estimated in twin studies to be around 74% [48], ADHD prevalence cannot be explained by genetic factors alone [48]. Several environmental factors, such as food additives/diet, lead contamination, cigarette smoke, alcohol, [49, 50], and exposure to environmental pollutants during pregnancy have been established as significant risk factors for developing ADHD [51]. Epidemiological studies have strongly implicated air pollution in the aetiology of neurodevelopmental disorders, including ADHD, although there is no definitive evidence yet [52–55]. The association between cumulative exposure to air pollutants from birth to diagnosis, particularly particulate matter of <10 *μ*m (PM_10_) and nitric dioxide (NO_2_), and childhood ADHD was nevertheless clearly demonstrated in a 10-year study (2003-2012) that tracked 8936 infants in the National Health Insurance Service-National Sample Cohort (2002-2012). Here, ADHD risk was increased by a factor of 2 to 3 when air pollutant concentration was increased by 1 *μ*g/m^3^ [52].

Genome-wide association studies (GWAS) have identified a large number of genes with moderate effects that are involved in ADHD susceptibility [56, 57]. Hawi et al. have recently identified ten ADHD candidate genes for which additional evidence such as meta-analyses, large-scale linkage studies, animal model research, and GWAS exists [58]. Most of these genes appear to be involved in synaptic transmission (*SNAP25*, *NOS1*, *LPHN3*, and *GIT1*), in monoaminergic function (dopamine and serotonin transporters and D4, D5, and 5-HT1B receptors) [58], or in the catecholaminergic system (such as DRD4 and DRD5 dopamine receptor genes, dopamine transporter gene, and dopamine beta-hydroxylase gene, which catalyzes conversion of dopamine to norepinephrine), and appear to play a crucial role in the appearance of disease [57, 59]. Alterations in these systems are mirrored in animal models of ADHD that include exposure to environmental chemicals during development. For instance, gestational and lactational exposure to BPA has been demonstrated to affect the offspring's brain development and dopaminergic system functioning [60]. Maternal exposure to BPA was also associated with both hypo- and hypermethylation of CpG islands in several loci in foetal mouse brains [61]. More recently, DasBanerjee et al. compared molecular changes in brain circuits involved in ADHD in both a genetic model of ADHD based on the spontaneously hypertensive rat (SHR) and a PCB-based model of ADHD [51]. Probing 218 unique genes considered highly relevant to ADHD or epigenetic gene regulation, expression levels of *Gnal*, *COMT*, *Adrbk1*, *Ntrk2*, *Hk1*, *Syt11*, and *Csnk1a1* were altered in both SHR rats and PCB-exposed SD rats, whereas *Arrb2*, *Stx12*, *Aqp6*, *Syt1*, *Ddc*, and *Pgk1* expression levels were changed only in PCB-exposed SD rats. Impaired expression levels of genes Oprm1, Calcyon, calmodulin, Lhx1, and Hes6 were only observed in SHRs. Epigenetic genes Crebbp, Mecp2, and Hdac5 were significantly altered in both models. This study demonstrated for the first time that environmental exposure to BPA can affect different sets of genes in two different models of ADHD inducing similar disease-like symptoms [51].

From an epidemiological point of view, analysis of di-2ethylhexyl phthalates in urine from pregnant women allowed the determination of a monotonic association between prenatal exposure to phthalates and increased risk factor of ADHD in a population-based nested case-control study conducted on the Norwegian Mother and Child Cohort [62]. Moreover, existing evidence that coexposure to socioeconomic disadvantage and air pollution in early life significantly increases the risk of adverse neurodevelopmental outcomes has been strengthened by a recent study in which nonsmoking African-American and Dominican pregnant women in New York City and their children were followed between 1998 and 2006 [55]. The authors demonstrated that children with high prenatal PAH exposure (high adducts) generally presented more symptoms of ADHD (higher scores) than those with low PAH exposure [55].

### 3.2. Autism Spectrum Disorder (ASD)

Autism is a complex heterogeneous neurodevelopmental disease with severe behavioural impairments (e.g., reduced social interaction, impaired communication, and repetitive behaviours) clustered under the term autism spectrum disorders (ASD) [63, 64]. According to estimates from CDC's Autism and Developmental Disabilities Monitoring (ADDM) Network, about 1 in 59 US children have been identified with ASD in 2018, versus 1 in 166 in 2004 [65]. In general, ASD results from abnormalities in brain development during pregnancy and after birth, which leads to an increase in brain volume at the age of 2 to 4 and to abnormalities such as microcolumns in the cerebral cortex [66]. ASD has a series of common psychiatric comorbidities (such as hyperactivity, anxiety, or epilepsy) as well as sleep disturbances and/or gastrointestinal disorders [64].

Although the aetiology of ASD remains largely unknown, it clearly originates from a transitory interruption early on in the brain development sequence [67]. The reasons underlying this interruption still remain to be fully elucidated. The strong heritability and genetic component of ASD were confirmed by the identification of 400 high-confidence genes involved in neuronal and cortical organisation, forming and maturing synapses, and neurotransmission and neuronal excitability within this set of syndromes [64, 68–71]. Environmental factors have recently emerged as additional important contributors to the aetiology and pathophysiology of ASD, and understanding these gene–environment interactions is now necessary [72]. Exposure to environmental chemicals (e.g., lead, methylmercury, PCBs, arsenic, fluoride, chlorpyrifos, DDT, tetrachloroethylene, PBDEs, and PAHs) or drugs (e.g., valproic acid (VPA), oxcarbazepine, and lamotrigine), particularly *in utero* or during early life, has been proposed to be involved in this process notably by affecting development of both the immune system and brain development [64].

VPA is an anticonvulsant and mood-stabilizing drug that appears to be the most widely studied of the drugs potentially linked to autism. Gadad et al. highlighted that “VPA enhances DNA demethylation, and while this mechanism may be useful for reverse hypermethylation in epilepsy and depression, it may also interfere with methylation processes necessary for healthy brain development” [72]. Children exposed to VPA during the first trimester of gestation present an increased risk factor of ASD (relative risk: 7.3; 95% CI: 4.4 to 12.2) [72]. Furthermore, exposing pregnant rat dams to VPA reduced cerebellar Purkinje cell numbers, cerebellar volume, and the number of neurons providing input to the inferior olive by 11%, 31%, and 9%, respectively [72, 73]. This concords with a significant decrease in Purkinje cell numbers observed in autistic individuals irrespective of age, sex, and cognitive ability postmortem [74]. As anatomical and functional maturation of the cerebellum takes place primarily after birth, and the cerebellum makes a crucial contribution to cognitive ability development [64], it is highly likely that exposure to environmental chemicals during the first months has a dramatic impact on brain functions later in life.

**Figure 2:**
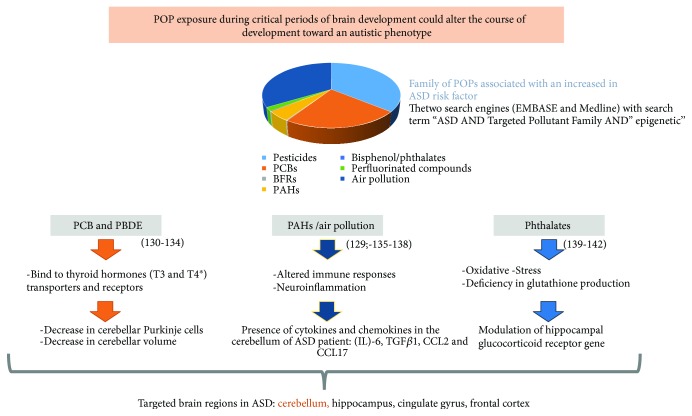
Family of POPs known to be associated with an increase in ASD – major brain regions involved in ASD which seems to be specific targets of POPs. The two search engines used as part of this review (EMBASE and Medline) with search terms “Autism Spectrum Disorders” AND “Targeted Pollutant Family” AND “epigenetic”.

Disruption of the endocrine, immune, and oxidative stress systems plays an important role in ASD pathogenesis. These three systems are all sensitive to POP exposure. Thyroid hormone (TH) analogues such as PBDE have been shown to bind to both “thyroid hormone transporters and receptors during critical periods of brain development may alter the course of development toward an autistic phenotype” [75]. Hertz-Picciotto et al. have shown that prenatal and early-life exposure to PBDEs in rodents results in a behavioural phenotype that mirrors ASD. This phenotype includes hyperactive spontaneous behaviours, deficiencies in memory and learning, and decreased stimulus-responsive capability [76]. Similarly, TH-disrupting PCBs inhibit or alter TH-regulated gene expression and promote brain development toward autistic phenotypes [75]. For example, exposure to OH-PCB 106 suppressed the TH-dependent dendritic development of cerebellar Purkinje cells in a culture model [77] (Figure 2). Behavioural impairment was transmitted to the F1 generation after F0 OH-PCB 106 exposure during the perinatal period [78]. Male offspring exposed in utero to OH-PCB 106 presented hyperactivity in both home cage and novel environments, whereas lactational exposure to OH-PCB 106 disturbed motor coordination and the ability of the animal to become accustomed to a novel environment [78]. By using whole-genome bisulphite sequencing in brain tissue and a neuronal cell culture model carrying a 15q11.2-q13.3 maternal duplication, Dunaway et al. found that PCB 95 exposure induced significant DNA hypomethylation over many autism candidate genes, significantly impacting expression of more than 1000 genes [79], demonstrating “the compounding effects of genetic and environmental insults on the neuronal methylome that converge upon dysregulation of chromatin and synaptic genes” [79]. These data suggest that exposure to POPs may cause Purkinje neuron abnormalities and are most probably critical determinants of a child's subsequent susceptibility to ASD.

Secondly, the immune responses play an important role in ASD pathogenesis. Neuroinflammation is a recurrent observation in almost all postmortem brain tissues collected from ASD patients [74, 80]. In the cerebellum, there is an accumulation of monocytes and macrophages together with increased cytokine and chemokine levels, such as IL-6, TGF*β*1, CCL2, and CCL17 [74]; increased cell surface major histocompatibility complex (MHC) proteins in microglia, and increase in glial fibrillary acidic proteins in astrocytes [74]. Autopsies conducted on urban dwellers exposed to acute air pollution revealed higher expressions of COX-2, a key enzyme involved in inflammation, in the frontal cortex and hippocampus compared to those observed in residents living in cities with better air quality [81], most probably due to ambient PM2.5 exposure [82]. Upregulation of COX-2 was also observed in both *in vitro* and *in vivo* models of benzo[a]pyrene exposure. Neonatal male Sprague-Dawley rats exposed to PM2.5 presented typical behavioural characteristics of ASD such as communication deficits, weak social interactions, and novelty avoidance during late adolescence and as adults [82]. At the highest exposure, mRNA expression and protein levels of five ASD candidate genes (SH3) and multiple ankyrin repeat domains 3 (Shank3) were significantly reduced in the hippocampus. Additionally, levels of the proinflammatory cytokines, IL-1*β*, IL-6, and TNF-*α* in the hippocampus and prefrontal cortex as well as markers of astrocytes and microglial cell activation were significantly increased upon PM2.5 exposure [82].

The third affected axis is detoxification. A high proportion of autistic children and their mothers have a deficiency in the production of glutathione [83], an antioxidant and a phase II enzyme involved in detoxification of environmental pollutants [84]. Low levels of glutathione, coupled with high production of homocysteine, increases ASD risk by a factor three [83]. This specific form of autism, associated with an X chromosome defect, appears four to five times more frequently in boys than in girls.

There is now evidence that phthalates, which increase oxidative stress in exposed mothers [85], directly induce abnormal epigenetic modifications in important foetal genes such as the hippocampal glucocorticoid receptor and adrenal steroidogenic acute regulatory protein [86]. Similarly, tracking two Chinese cohorts of mother-child diads with different levels of PAH exposure, Kalia et al. have demonstrated that PAH exposure could also induce epigenetic modifications. Lower exposure to PAHs was associated with beneficial effects on neurodevelopment as well as molecular changes related to healthier brain development. The authors hypothesize that “disruption in *LINE1* methylation during neurodevelopment may disturb normal neuronal plasticity and diversity controlled by *LINE1*, by increasing genomic instability, and interfering with gene expression” [87].

## 4. Early-Life Environmental Exposures and Epigenetic and Neurological Impairments in Adult/Elderly

AD and PD are two neurodegenerative diseases with many common characteristics. They affect large numbers of elderly people not only in high-income Western societies but also in intermediate- and low-income countries. Although ageing is the most obvious risk factor, and the number of people above the age of 60 is growing rapidly in a large majority of countries, the prevalence of both diseases has increased.

### 4.1. Alzheimer's Disease

AD constitutes a major health problem worldwide [88], representing 60-70% of senile dementia cases. Two main molecular actors have been recognized as critical players in AD progression, amyloid *β* peptide oligomers (A*β*o), and hyperphosphorylated tau (*τ*) protein. The *γ*-secretase BACE1 hydrolyzes amyloid precursor protein (APP) producing the principal form of A*β* peptide (A*β*_1-40_) and the more toxic A*β*_1-42_ at 90% and 10% abundance, respectively [89]. A*β* peptide aggregates into amyloid plaques which are one of two pathognomonic AD signs along with neurofibrillary tangles, as initially reported by Dr. Aloïs Alzheimer in 1906. However, A*β*_1-42_ forms oligomers before aggregating into amyloid plaques. These oligomers bind to synapses, altering their functions and plasticity [90]. It remains unclear whether A*β*o bind to a specific receptor or exert their neurotoxicity through direct interactions with the synaptic membrane [91]. Similarly, the relationship between A*β*o size/structure and level of neurotoxicity is poorly defined [92]. However, it is accepted that these A*β*o induce early synaptotoxicity and damage neuronal networks, causing memory impairment.

Tau is a neuronal signalling and axonal transport protein that is functionally modulated by phosphorylation [93, 94]. In AD, tau is hyperphosphorylated. This leads to tau helical filament formation, alterations of axonal transportations, and finally its aggregation into neurofibrillary tangles [95]. Hyperphosphorylation of tau in AD probably results from a disequilibrium between kinase (CDK5, GSK3*β*, ERK2, and/or other still unidentified kinases) and phosphatase activities. Tau hyperphosphorylation and aggregation is not specific to AD, but has been observed in numerous neurodegenerative diseases, collectively named tauopathies [94]. Although it has been extensively studied, the association between tau and A*β* peptide oligomers is still poorly understood; nevertheless, it appears that the pathological effects of A*β* peptide oligomers require the presence of intact tau [96].

As early as 2003, a relationship between occasional exposure to pesticides and cognitive impairments in men was observed [97]. This observation was independently reproduced in 2009 when a correlation between pesticide use in specific geographical areas and local AD and PD prevalence was reported. Additionally a clear increase in the number of cases of multiple sclerosis and suicides were reported [98]. In the US, however, detectable levels of the pesticide beta-hexachlorocyclohexane were found in 76% of PD patients versus 40% of controls and 30% of AD patients [99]. The second environmental factor investigated in epidemiological studies on AD risk was air pollution. In Taiwan, where the ageing rate is the second highest in the world, particle matter and ozone were found to have a significant impact on AD and vascular dementia [100]. Meta-analysis confirmed the correlation between pollution exposure and cognitive decline and dementia, although the duration of this exposure is still an open question [101]. Thirdly, the influence of heavy metal exposure on AD occurrence has been explored in epidemiological studies. Min and Min recently reported a correlation between cadmium levels in blood and AD mortality in 4064 US participants above the age of 60 [102]. A global meta-analysis on over 60 studies covering all forms of pollution identified air pollution, pesticides, and metals such as aluminium, silicon, and selenium as risk factors of dementia [103].

**Figure 3:**
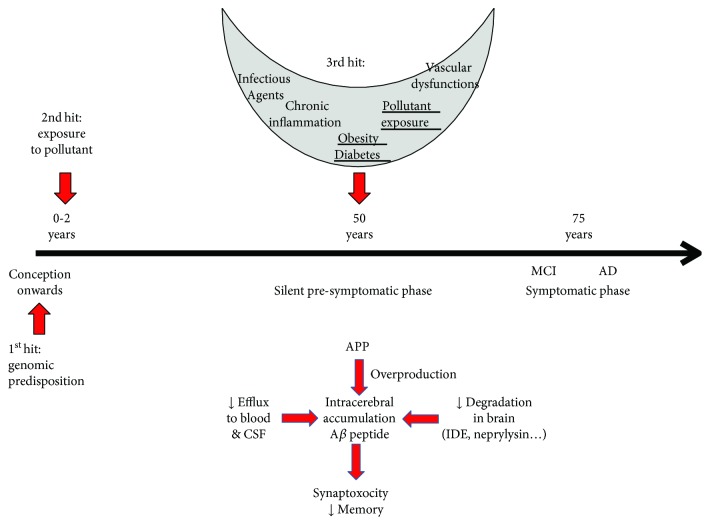
Three-hit hypothesis to explain the effect of an early exposure to pollutant on AD risk in late life. Exposure to pollutants in the neonatal period could impact epigenetic regulations. Late events could reactivate these regulations and affect A*β* accumulation or tau phosphorylation in the clinically silent phase which leads to MCI and clinically expressed AD. Mechanisms of A*β* accumulation in the brain: A*β* peptide could accumulate through 3 mechanisms: overproduction from APP, reduction of clearance through BBB, and inhibition of the various intracerebral degradation mechanisms. Increase of A*β*o lead to synaptic dysfunctions, neuronal death, and memory alterations.

Cellular and animal models have provided mechanistic insight into the effects of POP exposure. Pyrazole insecticides have the ability to activate *β*- and *γ*-secretases in samples derived from healthy individuals and patients affected by genetic forms of AD [104]. When deltamethrin and carbofuran were administered to mice, they induced spatial learning and memory deficits as well as reduced the expression levels of several memory-related synaptic proteins and induced tau hyperphosphorylation with activation of glycogen synthase kinase-3*β* and inhibition of protein phosphatase-2A [104]. Several studies using mice expressing mutant APP and presenilin genes showed that diesel and particulate matter increase A*β* production and amyloid plaque formation [105–107]. Similarly, cadmium interacts with A*β* peptide and favours its segregation from membranes and aggregation [108] while mercury increases A*β* peptide production and reduces neprilysin levels in neuronal PC12 cells [109]. Rats exposed to 3 ppm arsenic in drinking water from the neonatal period to the age of 4 months show cognitive deficits, higher *β*-secretase activity, and A*β* production in the brain, as well as higher A*β* clearance through the RAGE receptor [110]. This demonstrates that early exposure to pollutants induces effects observable in older animals. Early exposure to lead induced a similar cognitive decline, neuroinflammation [111, 112], A*β* peptide overproduction [113, 114], and tau hyperphosphorylation [115–118] in both mice and rats. Interestingly, Bihaqi et al. [119] observed a cognitive decline in rats only when exposed to lead as pups not as adults. Additionally, APP and BACE-1 overexpression were only observed in old rats, not in young animals. Furthermore, Dash et al. [118] observed an increase in miR-34c, which regulates tau gene expression. Lead exposure also induces short- and long-term reductions in expression of several other types of miRNA [120] and influences levels of Dnmts, MeCP2, and other proteins involved in histone modifications [121]. These findings support the hypothesis that epigenomic regulations could mediate the effects of early lead exposure on late AD-related events [122]. This could be extrapolated to the other pollutants as a two-hit hypothesis assuming that early exposure leads to epigenomic regulations which could bring about AD occurrence after a reactivation event such as renewed exposure to pollutants, obesity or diabetes, chronic inflammation, and vascular dysfunctions. When the initial genotype and gene-environment interactions in early life are further added into the equation, this produces a three-hit model, similar to that previously described by Daskalakis et al. [123] as summarized in Figure 3.

### 4.2. Parkinson's Disease

PD is widely considered as the second most prevalent neurodegenerative disease after AD. The age of latest onset is mostly over 60, although one in ten cases happen to be diagnosed before age 50. More seldom may PD affect people in their 40s and younger. In 2018, the European Brain Council estimated the number of people affected by PD regardless of race and culture to be over 6.3 million worldwide, 1.2 million of whom in Europe [124]. PD prevalence in industrialized countries generally stands at an estimated 1–2% of the population aged over 60 and increases to 3–5% in people above 85 years [125]. PD is defined as a motor progressive disorder of CNS characterized by degeneration of dopaminergic neurons in the substantia nigra, the brain region involved in both balance and movement. Loss of neurons goes with the formation of Lewy bodies (eosinophilic cytoplasmic inclusions containing aggregates of protein-like *α*-synuclein) in those remaining [122, 126]. Characteristic PD symptoms are tremor, rigidity (stiffness), slow movements (bradykinesia), and balance difficulties (postural instability) [124]. Motor symptoms appear at the stage where more than 60% of dopaminergic neurons are lost and 80–85% of dopamine content in the striatum is depleted [122, 127].

Although 5-10% of PD patients present with a monogenic form of the disease (thus suggesting a Mendelian type of inheritance), the majority of PD cases are sporadic [128], which is probably caused by the interaction of both genetic and environmental factors [129, 130]. No fewer than 6 genes, namely, SNCA (which encodes alpha synuclein), LRRK2 (leucine-rich repeat serine/threonine-protein kinase 2), *UCHL1* (ubiquitin carboxyl-terminal hydrolase isozyme L1), PARK2 (parkin), PINK1 (PTEN-induced kinase protein 1), and DJ-1 (protein DJ-1) have been associated with an autosomal dominant or recessive PD mode of inheritance [125, 129]. In addition to these genetic risk factors, environmental exposure to POPs is clearly regarded as a significant contributive factor in the etiology of this neurodegenerative disease alongside the ageing process [122]. For instance, causal linkages between exposure to pesticides (especially for dieldrin, paraquat, rotenone, and maneb), *α*-synuclein accumulation, and dopaminergic cell degeneration/apoptosis were established in individuals predisposed to *α*-synuclein accumulation for genetic or age-related reasons [131]. Meta-analysis based on case-control studies confirmed that pesticide exposure is significantly associated with gene alterations in PD (e.g., GST, PON-1, MDR1, and SNCA) and a higher risk of disease development [132]. Among the various molecular signalling pathways involved in PD, mitochondrial damage, energy failure excitotoxicity, protein misfolding, and aggregation - to name but a few - oxidative stress appears as one of the main contributors to environmental insult in PD [133]. For instance, MPP^+^, a metabolite of MPTP able to induce free radical injury, is selectively taken up by dopaminergic terminals and stored in neuronal mitochondria of the substantia nigra where it binds and inhibits complex I of the mitochondrial electron transport chain thereby inducing the oxidative stress often observed in PD patients [134, 135]. Depending on the environmental chemicals involved, oxidative stress could play a role in PD development: “i) the excess of peroxide formation caused by an increase in dopamine turnover, ii) the deficiency in glutathione which reduces the ability of the brain to phase out the free radicals which damage DNA, proteins and fat, iii) the increase in reactive iron that causes radical hydroxyl formation” [133, 136].

In sporadic PD, a definitive role of epigenetic modification in neurodegeneration has not yet been clearly established. Nevertheless, epigenetic mechanisms have been shown to lead to transcriptional silencing of genes involved in ROS scavenging, such as SOD2, a gene encoding for manganese superoxide dismutase [137, 138]. Exposure to pesticides such as dieldrin and paraquat resulted in histone acetylation [139]. Ammal Kaidery et al. suggested that after neurotoxic insult, histone acetylation could represent a key epigenetic modification in dopaminergic neuronal cells [139]. This hypothesis is supported by several studies which firstly showed that in dopaminergic neuronal cells, exposure to dieldrin induced a time-dependent increase in core histone acetylation [140]. In parallel, an induction of histone H3 acetylation associated with a decrease in total HDAC activity was also observed in N27 dopaminergic cells treated with paraquat [141].

Pesticide exposure during pregnancy or early life may determine the progressive damage of the substantia nigra years before the onset of clinical parkinsonism, as well as increase vulnerability to a second environmental factor (two–hit model) [142]. Dopaminergic cell loss and decreased dopamine levels were amplified when a rodent exposed to paraquat or maneb in early life was rechallenged as an adult, suggesting that developmental neurotoxin exposure enhanced the adult's susceptibility to a repeated toxic insult [143]. Similarly, prenatal exposure of pregnant C57BL/6J mice to PQ (0.3 mg/kg) or maneb (1 mg/kg) altered the development of the nigrostriatal system in the pups and enhanced their vulnerability to neurotoxins later in life (Barlow et al., 2004).

Recent epidemiological studies suggest that exposure to various environmental pollutants such as heavy metals (lead, manganese, as described in Chin-Chan et al. [122, 135]), solvents, and ultrafine particle matter (UFPM) may also increase the risk of developing PD. Nevertheless, very little is currently known about adverse effects of air pollutants on this neurodegenerative disease. Recent studies have however associated particulate exposures to brain pathologies like PD [144]. By studying children and young adults living in cities with high levels of air pollution and who died suddenly, Calderón-Garcidueñas et al. pointed out that brains of young people chronically exposed to air pollution showed an accumulation of alpha-synuclein, suggesting that exposure to air pollution should be considered a risk factor of PD [145]. Ten years later, the link between long-term exposure to traffic-related air pollution and PD was finally demonstrated [144]. The authors found that ambient air pollution from traffic sources was associated with risk of PD, with a 9% higher risk (95% CI: 3, 16.0%) per interquartile range increase (2.97 *μ*g/m^3^) in modeled NO_2_. For participants living for ≥20 years in the capital city, ORs were larger (OR = 1.21; 95% CI: 1.11, 1.31) than in provincial towns (OR = 1.10; 95% CI: 0.97, 1.26), whereas there was no association among rural residents [144].

## 5. The (Potential) Role of Epigenetics

### 5.1. Epigenetics as a Key Link between the Genes, Environment, and Phenotype

DNA methylation is probably the most intensely studied epigenetic mechanism. This endogenous genomic DNA modification is simply the covalent bonding of a methyl group to the 5-position of cytosine in CpG dinucleotides (5mC) [146–149]. DNA methylation has an established role orchestrating temporal as well as cell- and tissue-specific gene expression patterns throughout normal development as well as in adapting an individual to its environment [146, 147, 150]. Changes in DNA methylation appear to follow two clearly delineated paradigms, either (i) discrete hypo- and hypermethylation associated with functional effects, and clear molecular mechanisms, or (ii) a more subtle, complex, process where small (<10%) methylation changes are associated with disease phenotypes [151].

There are a series of reports examining the effect of maternal environment, through epigenetic mechanisms, altering transcription profiles and altering offspring neurodevelopment [152–155]. It is well established that modulation of the maternal hypothalamus pituitary adrenal (HPA) axis response to environmental challenges is able to strongly impact the offspring's neurodevelopment. The HPA axis is extraordinarily plastic and susceptible not only to parental care received in early life but also to parental stress-associated behaviours. Mechanistically, this has been shown to act through epigenetic regulation of genes such as the glucocorticoid receptor (GR), directly impairing lifelong neuroplasticity in highly sensitive brain areas like the hippocampus as well as lifelong changes in endocrine stress response [153, 156].

When molecular mechanisms were investigated, it was demonstrated that changes in Gr expression were due to methylation of 2 CpG dinucleotides in the Gr1_7_ promoter [154, 155], part of the complex 5′ gene structure responsible for tissue-specific regulation of Gr levels. Furthermore, expression of associated Gr1_7_ transcripts varied as a function of maternal care levels provided to a litter during early postnatal life [157]. High maternal care leads to rapid demethylation of this region postnatally, while methylation levels remain high if pups receive poor care. Although mechanisms leading to this rapid demethylation remain unknown many years later, these changes in methylation imply that poor care leads to a lower number of Gr and an increased response to stress.

These data clearly demonstrate that epigenetic processes directly “annotate” cues taken from the social environment during early life onto the genome. These lifelong effects are apparent not only on gene expression but also on epigenetic marks that influence the HPA axis stress response in adulthood. While somewhat outside the scope of this review, these data are among the few that clearly demonstrate a link between early environmental exposure, DNA methylation, and determination of a long-term phenotype.

Although these data are from rodents and remained limited in humans, it has been shown that transcriptional mechanisms are largely identical in the two species, and the human *GR1F* promoter and exon are orthologous to the rat 1_7_ promoter and exon [158–160]. Using tissue from the McGill Brain Bank, McGowan et al. examined *GR1F* promoter expression and methylation [152]. When both a history of family dysfunction and childhood adversity were taken into consideration, there was a clear link to an altered HPA stress responses and an increased risk for suicide [161], although our data, based on samples from the Netherlands Brain Bank, suggested that this was not the case [162, 163].

### 5.2. Epigenetics of Brain Development

Traditionally, epigenetic DNA methylation has been focussed on 5mC. However, there is an alternative, less abundant, modified form of cytosine, 5-hydroxymethylcytosine (5hmC). 5mC is oxidised by ten-eleven translocation (TET) enzymes, to form 5hmC [164, 165]. This is the first step of the active demethylation of 5mC to C via 5hmC, 5fC, and 5caC. Initially, 5hmC was thought to play a role in pluripotency and developmental reprogramming [166, 167]. Interestingly, 5hmC levels appear to be very poorly maintained in rapidly proliferating cells [168] and highly abundant in nonproliferating cells such as the CNS [169]. In cerebellar Purkinje cells, 5hmC levels are ~40% of 5mC levels compared to ~10% elsewhere in the body [164].

Although 5hmC is an intermediate in the active 5mC demethylation pathway, it is also a stable epigenetic mark. Within the brain tissue, 5hmC appears to play a role in differential gene splicing with differential methylation observed at GT-AG boundaries [170] and has a positive correlation with human cerebellum development [171], neurodevelopmental transcriptor factor regulation [172], and regulation of neuronal activity [164, 173, 174]. As evidence grows that 5hmC plays a role in neurodevelopment, intuitively, it will also be associated with neurodegenerative diseases. Although the data are somewhat preliminary, it has already been shown that oxidative stress can induce the oxidisation of 5mC to 5hmC [175], and the differences were in established stress-related genes [176]. Furthermore, differential 5hmC levels have been reported for Rett syndrome, ASD, and Alzheimer [177–179].

### 5.3. Transgenerational Epigenetic Effects or Epigenetic Inheritance?

It is important to distinguish between epigenetic inheritance and transgenerational epigenetic effects because of frequent misinterpretation of both expressions. Transgenerational epigenetic effects refer to transmission of epigenetic marks such as DNA methylation from gametes of an exposed individual to their offspring for one generation, whereas genuine epigenetic inheritance requires that both methylation and (patho-)physiological phenotypes are transferred across several generations, depending on exposure period. Environment-induced changes are referred to as *transgenerational epigenetic effects* when they occur in the pregnant adult female organism (F0), the first generation of offspring (F1), or the second generation of offspring (F2), because the F0 adult, the F1 foetus, and the primordial germ cells (PGCs) that will eventually go on to produce the F2 generation are all concurrently exposed during gestation. Only subsequent affected generations (F3, F4,…) can be considered as evidence of *epigenetic inheritance*. Obviously, if maternal exposure was *prior* to pregnancy, and for paternal exposure, proof of epigenetic inheritance starts one generation earlier [180, 181].

A significant example of epigenetic inheritance after exposure to a low molecular weight chemical product is the common dicarboximide fungicide vinclozolin which has lifelong sex-specific effects [182]. In males, differences in apoptosis and spermatocyte number and motility were maintained until F4 in adult males. This was further expanded to prostate abnormalities in F1-F4 males after exposure of only F0 females [183]. Transcriptional differences were observed in Sertoli cells from multiple generations [184]. However, female-specific effects included severe pregnancy anaemia in F1–F3 females and ovarian disease [185]. Changes in anxiety behaviour were sexually dimorphic [186]. While the *Gr* studies in the previous section were able to identify clear molecular mechanisms and individual CpGs, transcriptomic analyses performed in the vinclozolin model identified affected genes involved in chromatin remodelling and DNA methylation (e.g., Dnmt3a, Dnmt1, Dnmt3L, and Ehmt1) clearly implicating epigenetic processes.

These results obtained with vinclozolin brought forth a series of studies focussing on paternally transmitted phenotypes induced by endocrine-disrupting chemicals (EDCs) using somewhat similar paradigms to assess transgenerational effects of chemicals such as TCDD (2,3,7,8-tetrachlorodibenzo[para]dioxin), DEET (N,N-diethyl-meta-toluamide), BPA (bisphenol-A), DEHP (bis(2-ethylhexyl)phthalate), DBP (dibutyl phthalate), DDT (dichlorodiphenyltrichloroethane), and methoxychlor. Changes included prostate and kidney disease, mammary tumours, abnormalities in the immune system, neurological and behavioural effects, reproductive effects, altered mate preference, and obesity, although some effects were maternally transmitted [187–191]. Moreover, there are now data from several independent groups reporting epigenetically inherited phenotypes after endocrine-disrupting chemical (EDC) exposure (e.g., [192, 193]). Given the wide range of chemicals cited here, we suggest that long-term and transgenerational effects of persistent organic pollutants are not a phenomenon limited to a small number of compounds, but rather a general cocktail phenomenon.

### 5.4. What Does It Take to Generate a Latent Phenotype?

In a manner orthologous to POP exposure, early-life exposure to stress during critical periods of brain development has a lifelong effect on both emotional and cognitive functioning and behaviour [194] that acts via epigenetic changes such as DNA methylation and chromatin modifications [195]. In this paradigm, there is clear, concurring, human evidence that traumatic early-life stress is a major psychiatric disease risk factor. Work on environmental control of the HPA axis, stress phenotypes, and resultant psychopathologies has led to the development of a series of neurodevelopmental and behavioural models that try and explain the environment-phenotype link.

The initial model was a cumulative model, whereby stressors accumulate throughout life, and when cumulative stress passes a given threshold, the risk of psychopathology is significantly increased [196]. As the DOHaD model gained adherents, it was proposed that negative early-life experience would enter into the developmental match/mismatch hypothesis. Here, the early-life environment induces stable DNA methylation changes that are part of a “predictive adaptive response” [197]. This epigenetic plasticity corresponds to the individual adapting to its unique “predicted” lifelong environment. A subsequent mismatch between the adapted phenotype and the later life environment was then thought to underlie the increased risk of psychopathology.

Neither of these models considered the role of an individual's genetic make-up, an essential determinant of differential susceptibility to a given environmental influence. Belsky and Beaver suggested that a genetic susceptibility should be considered together with the environmental context and that “vulnerability in one environment may actually constitute an adaptive benefit in another environment” [198]. It has become clear over the years that there are strong gene-environment interactions. In particular, genetic variation in HPA-axis genes moderates the long-term effects of stress, conferring either an adaptive advantage or a risk for psychiatric disorders. The two key genes regulating the HPA axis are the glucocorticoid and mineralocorticoid receptors (GR and MR, respectively). The GR promoter region contains a minimum of 12 known SNPs [158], many of which are in linkage disequilibrium and make commonly accepted haplotypes [199] that play a significant role in controlling levels of GR transcripts [159, 160, 200, 201]. When the role of GR genetic and epigenetic factors on the HPA axis response to a psychosocial stressor was investigated, it was found that the physiological response was an “exquisite mix of pre-determined (genetic) and environmentally influenced (epigenetic) factors” [202].

**Figure 4:**
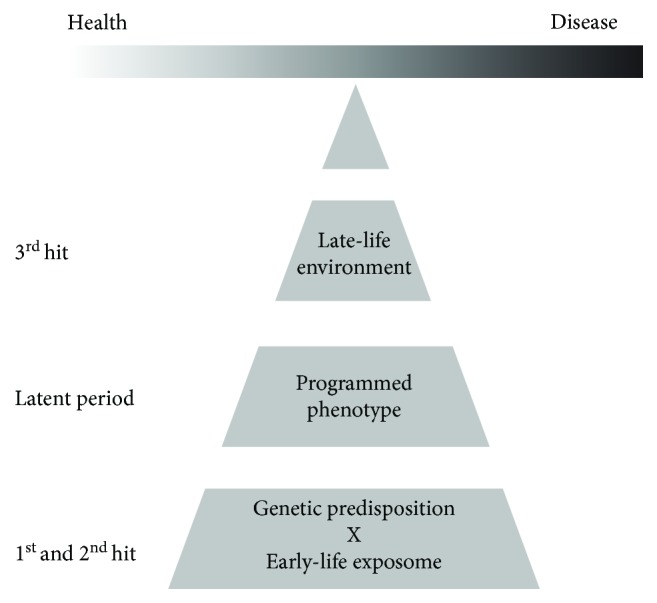
The three-hit hypothesis as initially presented by Daskalakis et al. in 2013. The first two hits in early life provide an epigenetically programmed quiescent or latent phase. Subsequent exposure to a third hit can “swing the balance” towards health or disease. Adapted from [123].

This knowledge has now been brought together in the most recent “three-hit” model (Figure 4) that was proposed by Daskalakis et al. in 2013 [123] as a testable framework that considers the genetic factors as a first “hit.” It has an immediate interaction with the early-life environment, the second “hit.” Together, these first two hits produce a quiescent or latent epigenetic phenotype that has become susceptible in later life to a third environmental “hit” after which the increased risk of psychiatric symptoms is crystallised. However, if the individual is exposed to a different environment in later life, they would be expected to remain healthy.

## 6. Conclusion

**Figure 5:**
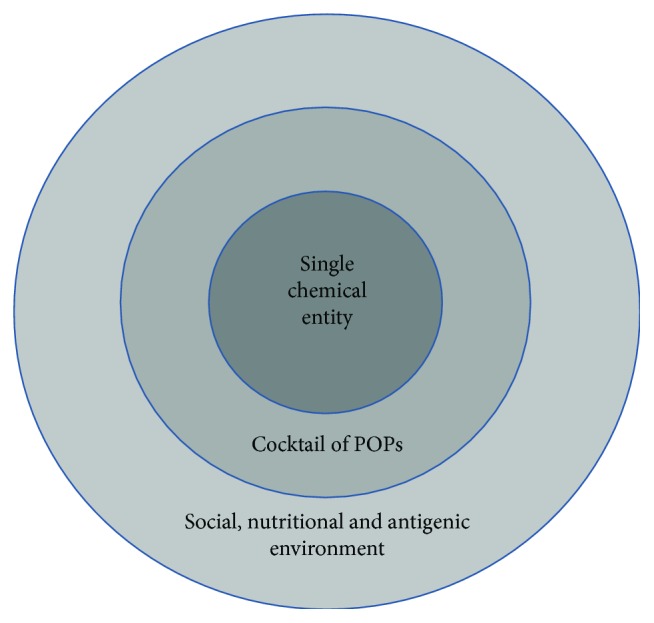
The concentric circle model of the early-life exposome. The complete early-life environment should be considered as generating the first two “hits” in the proposed “three-hit” model.

This review has covered long-term neurodevelopmental and neuropsychiatric effects of exposure to persistent organic pollutants. Evidence is growing that exposure to low doses of POPs during a critical developmental period can have a wide range of neurodevelopmental consequences. Importantly, if neurodevelopmental processes are halted, inhibited, or in some way altered, there is little potential for later repair, with potentially permanent consequences. There is an unfortunate preponderance of literature focussed on single-molecule exposure models. However, within these models, trends are starting to emerge. HAP would appear to be linked to ADHD; PCBs have been linked to ADHD and ASD, while both pesticides and air pollution have been linked to all four diseases. By comparing the POP-exposure literature with that of stress-HPA-axis-Psychopathology studies we can see many parallels. However, our comparison also highlights many significant gaps in our current knowledge. The two search engines used as part of this review (EMBASE and Medline) with search terms “Disease Name AND Targeted Pollutant Family” AND “epigenetic” have shown that for the time being, even though a certain number of studies reveal neurotoxic effects of organic pollutants in animal models, few of them happen to take the disease dimension into consideration, and none take the complete early-life exposome into consideration. Furthermore, there was a dearth of interest in the mechanistic role of epigenetics with very few published studies. Although the literature appears to suggest that both pesticides and PCBs play a role in the emergence and development of neurodevelopmental diseases, the lack of knowledge available about the other pollutants in the complex mixtures we are exposed to, particularly in air pollution, raises the question of cocktail effects rather than single responsible pollutants. Similar profiles can be seen for neurodegenerative diseases such as Alzheimer's disease with pesticides and PAHs. In this case, both pesticides and PAHs have been shown to be clear environmental risk factors in the appearance of the disease. We suggest that the environmental exposure is, however, somewhat more complicated. While there are clear effects from single chemical entities or classes, this is not a realistic model of the human situation. Regardless of the disease targeted, exposure under “real-life” conditions involves low-dose, long-term exposure to multiple environmental chemicals. Furthermore, such environmental POP exposure will be co-incident with many other forms of early-life adversity. For example, exposure to polluted air is strongly associated with both SES and the well-established negative health effects of low SES. We propose a concentric circle model of the overall early-life exposome (Figure 5). In the case of exposure to a particular POP, this needs to be placed into the context of the cocktail of other POPs to which the individual is exposed. Both the inner circle POP and the cocktail of POPs in the next circle out will have clear epigenetic effects, particularly on DNA methylation as outlined in this review. Furthermore, this exposure, together with all the other aspects of the early-life environment, will act together. There will be a complex interplay between the individual's genome, their plastic epigenome that is moulded by the complete early-life exposome, and their exposure to POPs. Taken together, these three elements will generate the latent “disease-free” phenotype that is waiting for the final trigger to induce neurological disorders many years later.

## Abbreviations

ASD: Autism spectrum disorder
ADHD: Attention-deficit hyperactivity disorder
ALS: Amyotrophic lateral sclerosis
AD: Alzheimer disease
SES: Socioeconomic status
HPA: Hypothalamus pituitary adrenal axis
EDCs: Endocrine-disrupting chemicals
TCDD: 2,3,7,8-Tetrachlorodibenzo[para]dioxin
DEET: N,N-Diethyl-meta-toluamide
BPA: Bisphenol-A
DEHP: Bis(2-ethylhexyl)phthalate
DBP: Dibutyl phthalate
DDT: Dichlorodiphenyltrichloroethane
GR: Glucocorticoid receptor
PCB: Polychlorinated biphenyls
PCDDs: Polychlorodibenzo-para-dioxines
PCDFs: Polychlorodibenzofuranes
BFRs: Brominated flame retardants
HBCDD: Hexabromocyclododecane
TBBP-A: Tetrabromobisphenol A
PBDEs: Polybrominated diphenyl ethers
PBB: Polybromobiphenyls
PAHs: Polycyclic aromatic hydrocarbons
SVHC: Substances of very high concern
ECHA: European chemical agency
EFSA: European Food Safety Authority
BBB: Blood-brain barrier
PAH: Polycyclic aromatic hydrocarbon
VPA: Valproic acid
OH-PCB: Hydroxylated polychlorinated biphenyl
POP: Persistent organic pollutant.
